# Incorporation of arabinose-CTP and arabinose-UTP inhibits viral polymerases by inducing long pauses

**DOI:** 10.1016/j.jbc.2025.111027

**Published:** 2025-12-09

**Authors:** Ziyang Xiao, Arnab Das, Abha Jain, Thomas K. Anderson, Craig E. Cameron, Jamie J. Arnold, David Dulin, Robert N. Kirchdoerfer

**Affiliations:** 1 Institute for Molecular Virology, Center for Quantitative Cell Imaging, Department of Biochemistry, University of Wisconsin-Madison, Madison, Wisconsin, USA; 2 Department of Physics and Astronomy, and LaserLaB Amsterdam, Vrije Universiteit Amsterdam, Amsterdam, The Netherlands; 3 Department of Microbiology and Immunology, University of North Carolina School of Medicine, Chapel Hill, North Carolina, USA

**Keywords:** SARS-CoV-2, poliovirus, viral polymerase, single-molecule biophysics, magnetic tweezers, cryo-EM, nucleoside–nucleotide analog, plus-stranded RNA virus

## Abstract

Key to supporting human health in the face of evolving viruses is the development of novel antiviral drug scaffolds with the potential for broad inhibition of viral families. Nucleoside analogs are a key class of drugs that have demonstrated potential for the inhibition of several viral species. Here, we evaluate arabinose nucleotides (ara-NTP) as inhibitors of the severe acute respiratory syndrome coronavirus 2 and poliovirus polymerases using biochemistry, biophysics, and structural biology. Ara-NTPs compete poorly with their natural counterparts for incorporation into RNA by viral polymerases. However, upon incorporation, ara-NMPs induce long polymerase pausing during both severe acute respiratory syndrome coronavirus 2 and poliovirus polymerase RNA elongation. Our studies suggest that following ara-NMP incorporation, additional nucleotide incorporation is inhibited at the catalytic step.

The constant evolution and emergence of viruses to cause human infections represents a moving target for the design of novel antiviral drugs. Nucleotide analogs are a class of antiviral drugs frequently used to treat viral infections. These analogs have been used to treat RNA virus infections such as those caused by HIV-1 (1), hepatitis C virus (2), influenza virus (3), and coronaviruses. Nucleotide analogs have also found use in treating both infections by DNA viruses like herpesviruses as well as cancer (4, 5).

The emergence of severe acute respiratory syndrome coronavirus 2 (SARS-CoV-2) in 2019 to cause coronavirus disease 2019, resulted in a global pandemic. While the pandemic emergency has subsided, SARS-CoV-2 continues to circulate and evolve (6, 7). Though vaccines against SARS-CoV-2 are highly effective at preventing infection or mitigating severe symptoms (8), antiviral drugs are necessary to counter breakthrough infections and aid those who are unable or unwilling to be vaccinated. Nucleotide analogs typically target viral polymerases. The RNA-dependent RNA polymerase of SARS-CoV-2 is composed of a catalytic subunit, nsp12, which contains conserved polymerase motifs, as well as nsp7 and nsp8 replication factors that enable the binding of RNA (9). The nsp12 subunit is among the most conserved proteins encoded within the viral genome (10) making it a choice target for drug discovery efforts.

Two nucleotide analog antiviral drugs have been used to treat SARS-CoV-2 infections. Remdesivir is the only Food and Drug Administartion approved nucleotide analog for the treatment of severe coronavirus disease 2019 and has been found to reduce mortality and progression to mechanical ventilation (11). Remdesivir acts as an ATP analog and is readily incorporated into nascent RNA chains by the SARS-CoV-2 polymerase (12). The incorporation of remdesivir leads to delayed pausing, where the polymerase continues to extend the nascent chain a further three nucleotides whereupon the 1′-cyano group of remdesivr encounters a steric clash with nsp12 Ser861, creating a translocation barrier (12, 13). The inhibition of the SARS-CoV-2 polymerase by remdesivir can be overcome with physiological nucleotide concentrations indicating that this nucleotide analog induces polymerase pausing but not termination (14). A second nucleotide analog, molnupiravir, has emergency use authorization. Studies of molnupiravir indicate that it reduces mortality and is well tolerated (15). Molnupiravir is a cytidine analog able to base pair with both adenosine and guanosine, inducing mutations into the genome upon subsequent rounds of RNA replication. Importantly, molnupiravir evades proofreading by the coronavirus exonuclease (16). While molnupiravir can induce lethal mutagenesis, the increase in potential viral diversity has raised concerns about the generation of new viral variants (17, 18). Additional studies have suggested possible genotoxic effects where metabolism of molnupiravir may lead to its incorporation into DNA though differing results have been obtained from *in vitro* and *in vivo* studies (19, 20).

To keep pace with evolving and emerging viruses and to diversify the mechanisms used to inhibit viral enzymes, it is necessary to discover and evaluate new antiviral drug scaffolds. Here, we evaluate the arabinose nucleotides, ara-CTP and ara-UTP, as inhibitors of both SARS-CoV-2 and poliovirus polymerases. Arabinose nucleotides are isomers of their corresponding natural nucleotides with an inverted stereochemistry at the C2′ position of the ribose sugar. Arabinose nucleotides are considered analogs of deoxy-cytosine triphosphate (dCTP) (21). Ara-cytosine (ara-C), also referred to as cytosine arabinoside and cytarabine, is used for the treatment of acute myeloid leukemia and acute lymphoblastic leukemia (22). Ara-C is phosphorylated upon entering cells to ara-CTP and competes with dCTP for incorporation into DNA by DNA polymerases. DNA polymerase extension from incorporated 3′ ara-CMP is very slow, and incorporation inhibits diverse DNA polymerases, including Pol-α, Pol-ε, and avian myeloblastosis virus reverse transcriptase as well as terminal transferases and DNA ligases (21, 23). Ara-thymidine (ara-T) and ara-uridine have similarly been shown to inhibit DNA polymerases by competing with deoxy-thymidine triphosphate for incorporation into DNA by DNA Pol-β (24, 25). Like ara-C, ara-T incorporation into DNA is dependent on its conversion to ara-TTP, which has a low efficiency when using cellular thymidine kinase (25). However, herpes simplex virus-1 and -2 express a viral thymidine kinase that much more readily converts ara-T into its active triphosphate, providing some selectivity for targeting herpesvirus-infected cells (25). Here, we demonstrate that ara-CTP and ara-UTP are inhibitors of viral RNA polymerases *in vitro*, where incorporation induces long pauses likely at the subsequent catalytic step.

## Results

### Incorporation of ara-NTP inhibits SARS-CoV-2 polymerase extension

**Figure 1:**
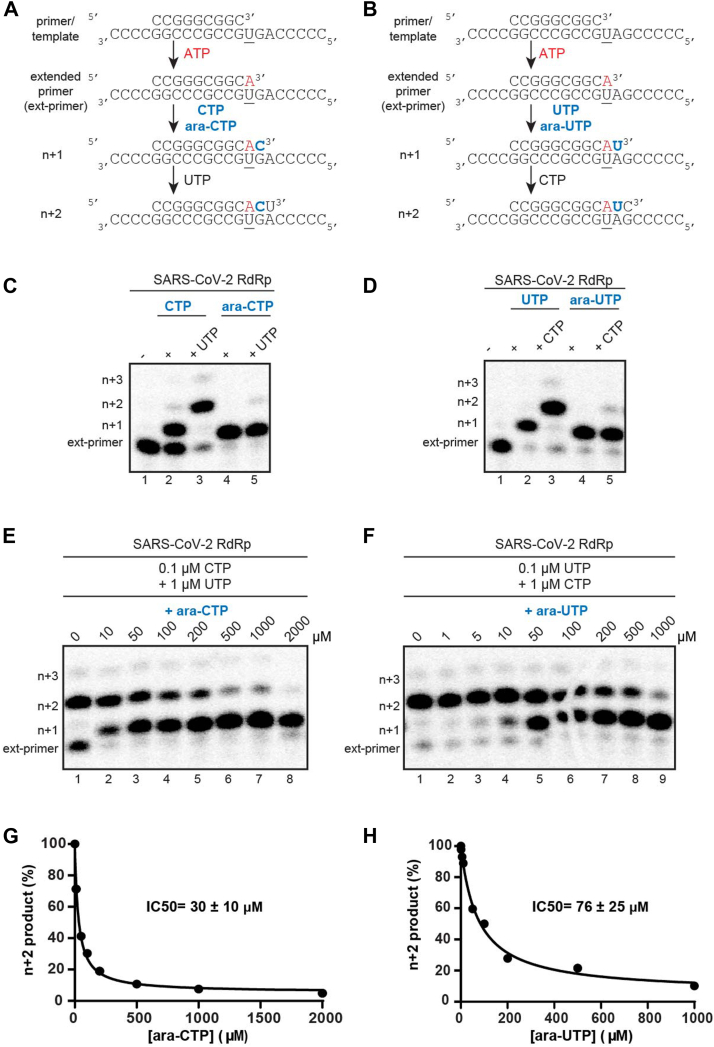
**Incorporation and competition of ara-NTPs**. *A* and *B*, schematic of the primer extension assay used to evaluate SARS-CoV-2 polymerase activity. *C* and *D*, incorporation and extension of ara-CTP and ara-UTP. SARS-CoV-2 polymerase–catalyzed incorporation of nucleoside triphosphates using CTP or ara-CTP and UTP or ara-UTP in the absence and presence of the next correct NTP, respectively. These incorporation experiments were independently repeated at least three times with similar results. *E*–*H*, competition of arabinose nucleotides with their natural counterparts. SARS-CoV-2 polymerase catalyzed nucleotide incorporation in the presence of increasing concentrations of (*E*) ara-CTP with 0.1 μM CTP and 1 μM UTP and (*F*) ara-UTP with 0.1 μM UTP and 1 μM CTP. *F*, the 100 μM band in the gel is broken because of cracked gel. Data were fit to a dose–response curve (*G* and *H*). The IC_50_ values for ara-CTP and ara-UTP under these conditions are 30 ± 10 μM and 75 ± 25 μM, respectively. SARS-CoV-2, severe acute respiratory syndrome coronavirus 2.

To evaluate arabinose nucleotides (ara-NTPs) as inhibitors of viral polymerases, we tested the ability of the SARS-CoV-2 polymerase complex to incorporate and extend from ara-CTP and ara-UTP. Single incorporation assays show that both natural NTPs and ara-NTPs are incorporated into primer RNAs by the SARS-CoV-2 polymerase. However, incorporation of either ara-CTP or ara-UTP prevents further extension in a primer extension assay performed at low NTP concentrations (0.1 μM NTP) (Fig. 1, *A*–*D*, S1*A*). This result shows that the inhibition of the SARS-CoV-2 polymerase is dependent on single nucleotide incorporation into the nascent RNA strand. We used this assay to measure the ability of each ara-NTP to compete with the corresponding natural nucleotide where extension halting at n + 1 indicates incorporation of ara-NTP and pausing and extension to n + 2 indicates incorporation of the natural NTP with subsequent extension (Fig. 1, *E*–*H*). Both ara-CTP and ara-UTP compete poorly with their corresponding natural nucleotides. The IC_50_ for ara-CTP is 30 μM in the presence of 0.1 μM CTP. Similarly, ara-UTP has an IC_50_ of 76 μM in the presence of 0.1 μM UTP. This indicates a strong preference by the SARS-CoV-2 polymerase to use natural nucleotides over their arabinose analogs.

**Figure 2:**
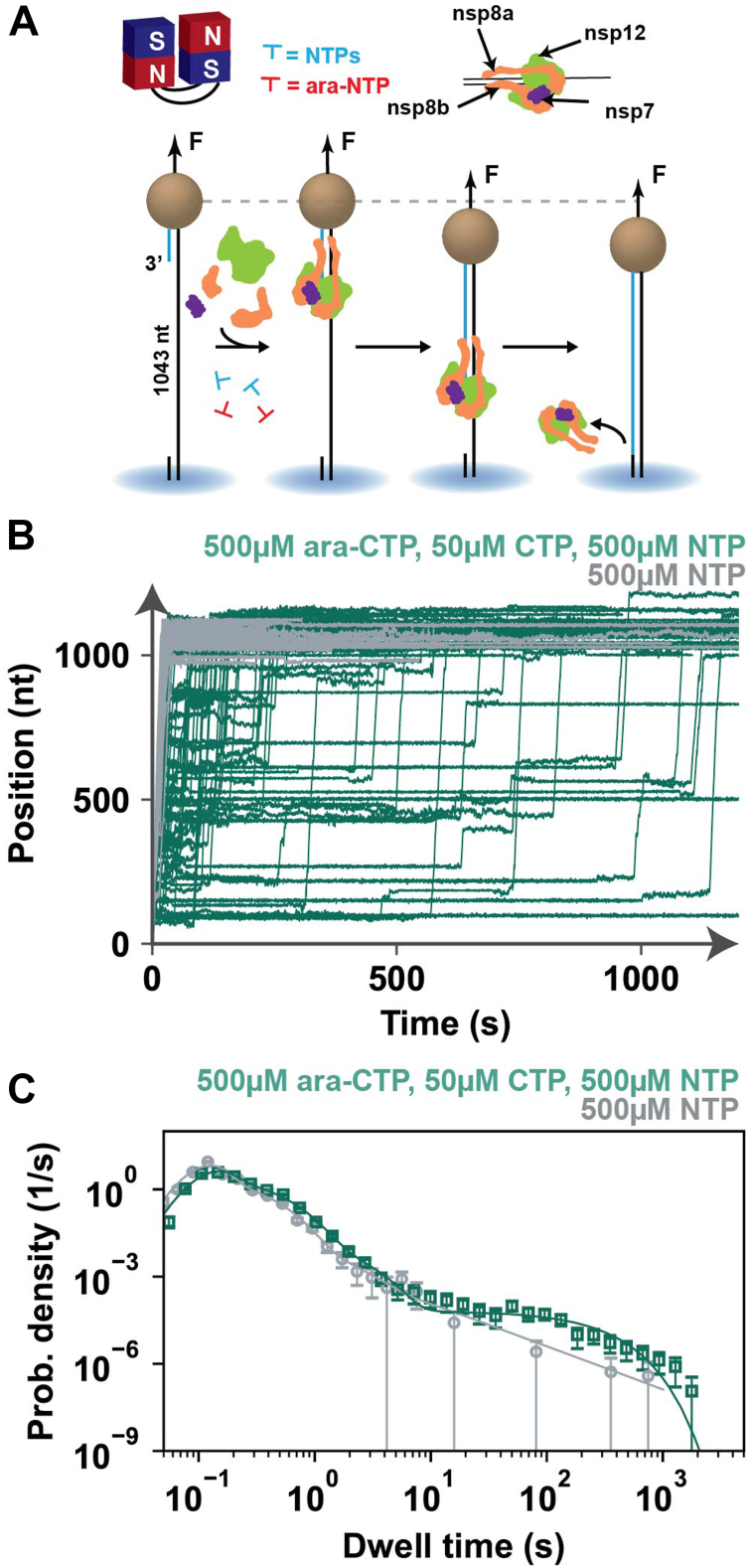
**ara-CTP incorporation induces long-lived, exponentially distributed pauses in SARS-CoV-2 RNA polymerase elongation dynamics**. *A*, schematic of the magnetic tweezer assay to monitor SARS-CoV-2 polymerase RNA synthesis activity. *B*, SARS-CoV-2 polymerase RNA synthesis activity traces for either 0 μM ara-CTP and 500 μM NTP (*gray*) or 500 μM ara-CTP, 50 μM CTP, and 500 μM other NTP (*dark green*). *C*, dwell time distributions extracted from the SARS-CoV-2 polymerase activity traces described with the same color code as indicated in *A*. The *solid lines* are maximum likelihood estimation (MLE) fits of the pause-stochastic model. The error bars are one standard deviation from 1000 bootstraps of the datasets. SARS-CoV-2, severe acute respiratory syndrome coronavirus 2.

To further investigate the mechanism of action of the ara-CTP nucleotide analog on the SARS-CoV-2 polymerase, we used high-throughput magnetic tweezers (Figs. 2, S2, S3) (14), which take advantage of longer RNA templates, and monitor ara-NTP incorporation in the presence of all natural NTPs, including the competing one, at higher nucleotide concentrations. While the single- and double-nucleotide incorporation studies above showed that the SARS-CoV-2 polymerase halts after ara-NMP incorporation, the higher nucleotide concentrations used in the magnetic tweezer experiments permit extension from incorporated ara-nucleotides. Here, a ∼1 kb ssRNA template was used to tether magnetic beads to a glass surface of a flow chamber using short, modified RNA oligonucleotides, which also provide free 3′ termini for polymerase extension (Fig. 2*A*). The polymerases elongated the primer in the presence of all NTPs with or without the nucleotide analog at an ara-CTP:CTP ratio of either 1:1 or 10:1. Polymerase extension shortens the length of the RNA tether through the conversion of the single-stranded RNA template into dsRNA. By following the vertical position of the magnetic bead in real time, we can monitor the position of the polymerase along the template with near single-base resolution and can derive the polymerase’s nucleotide addition dynamics (Figs. 2, S3, Table S1).

Previous studies of the SARS-CoV-2 RNA polymerase using magnetic tweezers have revealed rapid incorporation of nucleotides with evidence of two on-pathway polymerase states associated with slower nucleotide incorporation states as well as an off-pathway long-lived kinetic state assigned to backtracked polymerases (26). The slow nucleotide addition pathway has been suggested to represent a lower fidelity polymerase state (pause 1) and the very slow nucleotide addition pathway as representing polymerase extension from the products of nucleotide misincorporations (pause 2) (27).

In the presence of ara-CTP, we monitored an increase in the number of long-lived pauses interrupting the activity traces (Figs. 2, S3, Table S1), increasing the average RNA replication time while not affecting the mean product length. The unaffected product length in the presence of ara-CTP at higher NTP concentrations suggests that contrary to bulk biochemistry experiments above, ara-CTP incorporation by the SARS-CoV-2 RNA polymerase introduces strong polymerase pausing but does not act as a terminator. Previous work examining the potential of ara-UTP as an inhibitor of SARS-CoV-2 polymerase similarly found that ara-UMP incorporation does not result in termination and that ara-UTP is not a preferred substrate over natural UTP. However, the longer endpoint assays adopted in this previous study did not allow observation of either the prolonged pausing induced by ara-UMP incorporation or inhibition of SARS-CoV-2 polymerase primer extension (28).

We performed a dwell time analysis of the SARS-CoV-2 polymerase magnetic tweezer traces. Traces were scanned with a nonoverlapping 10-nt window to measure the amount of time polymerase took to incorporate 10 successive nucleotides (dwell times). The dwell time duration is the temporal signature of the biochemical process that dominated the 10-nt addition. The resulting dwell times are assembled into a probability density distribution represented in a log-binned histogram (Figs. 2, S3) (27). These distributions were then fitted using a stochastic pausing translocation model from which we derive the nucleotide addition rate, probabilities for polymerase entering a slow or very slow nucleotide addition pathway and backtracked states, as well as the exit rate of each nucleotide addition pathway (27, 26) (Table S1).

Both the slow and very slow nucleotide addition pathways (pause 1 and pause 2) are exponentially distributed. In the absence of ara-CTP, the long-lived pause is best described by a power law in the absence of ara-CTP, as previously reported for SARS-CoV-2 polymerase (14). In the presence of ara-CTP, the long-lived pause is best described by an exponential distribution, which we term the ara-CTP pause. The exponential distribution of the ara-CTP pause indicates that such a pause is exited through a single rate-limiting kinetic step with an exit rate k_ara-CTP_ = 0.005 s^-1^, that is, the ara-CTP pause has an average lifetime of ∼200 s (Table S1).

The nucleotide addition, slow and very slow nucleotide addition pathway’s exit rates were largely unaffected by the addition of ara-CTP as compared with the SARS-CoV-2 RNA polymerase with 500 μM NTPs. The slight difference observed in pause 2 exit rate at the 10:1 analog stoichiometry is likely because of the difficulty in fitting the pause 2 exponential when the ara-CTP pause becomes dominant in the dwell time distribution.

The strong pausing observed in the magnetic tweezers for ara-CTP and SARS-CoV-2 could be recapitulated in bulk biochemistry assays. In these assays, ara-CTP or ara-UTP is incorporated into primer–template pairs by the SARS-CoV-2 polymerase and then chased with 40 μM of each of the remaining NTPs. Over the course of the experiment, incorporated ara-NMPs are extended to a full-length product demonstrating escape from the strong polymerase pausing (Fig. S4). Despite SARS-CoV-2 polymerase’s ability to extend from both ara-CMP and ara-UMP incorporations, ara-CMP proved more difficult to extend from with significant inhibited product remaining after an hour, whereas ara-UMP could be extended to full-length product in 30 min. The lack of significant stalling beyond the incorporated ara-NMP supports the finding from the bulk biochemistry experiments above in that a single ara-NMP incorporation is sufficient to inhibit SARS-CoV-2 polymerase and differs from the nucleoside analog remdesivir, which pauses the SARS-CoV-2 polymerase after several additional nucleotide incorporations (12).

### Ara-NTPs bind the SARS-CoV-2 polymerase active site and are readily incorporated

**Figure 3:**
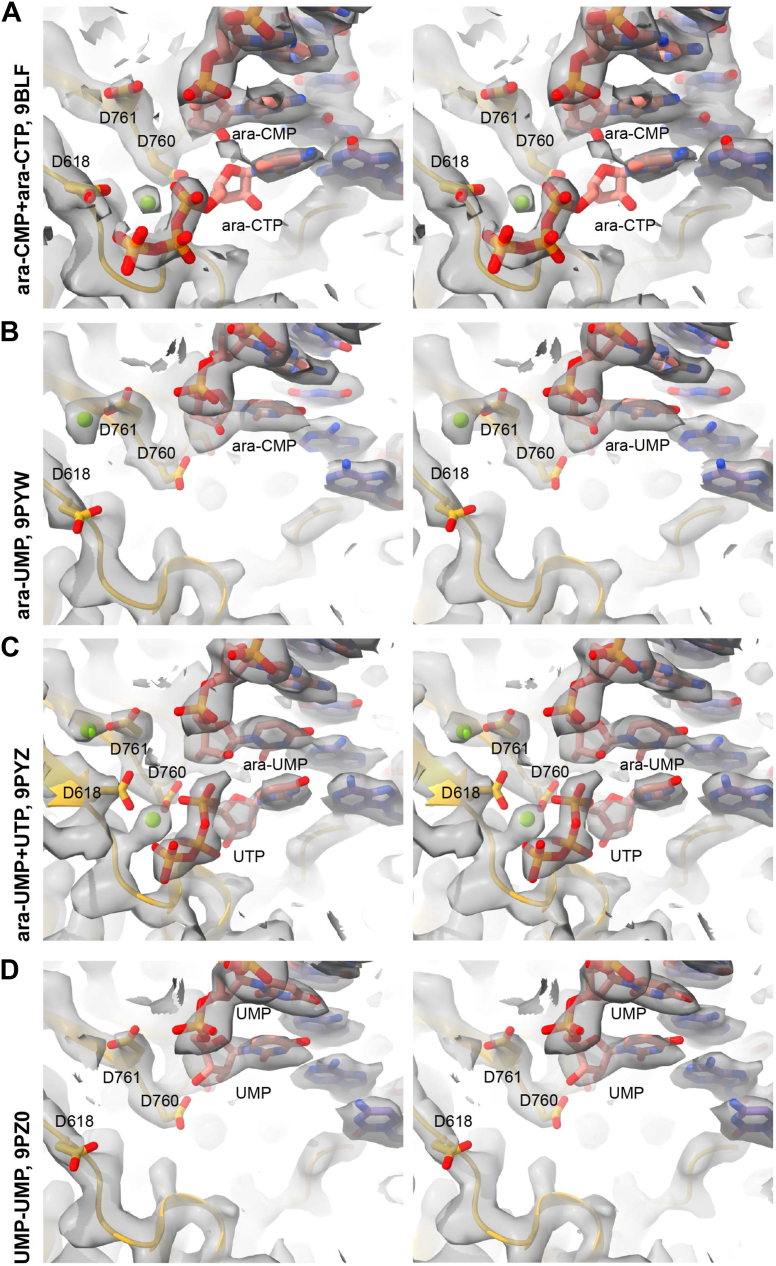
**Stereo images of determined structures in reconstructed density**. *A*, coordinate model (pdb_00009BLF) for the SARS-CoV-2 replication–transcription complex (RTC) with incorporated ara-CMP is shown in the corresponding reconstructed density (EMD-44654) isosurface contoured to 0.28. *B*, coordinate model (pdb_00009PYW) for the SARS-CoV-2 RTC with incorporated ara-UMP is shown in the corresponding reconstructed density (EMD-72038) isosurface contoured to 0.65. *C*, coordinate model (pdb_00009PYZ) for the SARS-CoV-2 RTC with incorporated ara-UMP and bound UTP is shown in the corresponding reconstructed density (EMD-72053) isosurface contoured to 0.65. *D*, coordinate model (pdb_00009PZ0) for the SARS-CoV-2 RTC with two incorporated UMP is shown in the corresponding reconstructed density (EMD-72054) isosurface contoured to 0.65. SARS-CoV-2, severe acute respiratory syndrome coronavirus 2.

**Figure 4:**
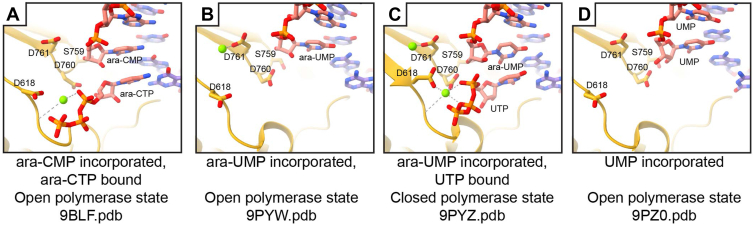
**SARS-CoV-2 polymerase active site poses for incorporated ara-NMPs**. *A*, coordinate model of the polymerase active site with incorporated ara-CMP with incoming ara-CTP. *B*, coordinate model of the polymerase active site with incorporated ara-UMP. *C*, coordinate model of the polymerase active site with incorporated ara-UMP with incoming UTP. *D*, coordinate model for the polymerase active site with two incorporated UMPs. S759, D760, and D761 correspond to the key residues within polymerase motif C. D618 is a conserved residue of polymerase motif A, which shifts position to close the RNA polymerase active site when a nucleotide is bound (37). SARS-CoV-2, severe acute respiratory syndrome coronavirus 2.

To further examine the mechanism of action of ara-NTPs against SARS-CoV-2, we used cryo-EM to determine several structures with incorporated ara-NMPs (Fig. 3, S5-8, Table S2). The 3.3 Å structure of the SARS-CoV-2 polymerase complex with incorporated ara-CMP shows good resolution for the nsp12 catalytic subunit as well as nsp7 and two nsp8 cofactors. We observe 30 base pairs of dsRNA and six single-stranded nucleotides of downstream template. The polymerase has incorporated one ara-CMP and has translocated to allow the binding of an incoming ara-CTP (Figs. 4, S5, S9). The density for the incoming ara-CTP is weaker than surrounding protein regions, but we can clearly position the nucleotide base and triphosphate. We also observe an ara-CTP bound to the N-terminal nidovirus RNA polymerase–associated nucleotidyltransferase (NiRAN) domain in a base-in conformation (Protein Data Bank [PDB] codes: 7ED5 (29) and 7UOB (30)) with H-bonds of Cys53 to the arabinose 2′OH and Tyr257 to the cytidine base. The binding of ara-CTP to the NiRAN appears to take advantage of the catalytic metals in the NiRAN active site as has been observed in several in-facing and out-facing NTPs (PDB codes: 6XEZ (31) and 7CYQ (32)). We determined a similar structure with an incorporated ara-UMP though without reliable density to model an incoming ara-UTP or NiRAN-bound nucleotide (Figs. 3B, S6, S9). The ara-nucleotides were modeled with C2′-endo sugar puckers, as has been suggested by previous structural studies (33, 34, 35, 36, 37). In both incorporated ara-NMP structures, the nucleotide analogs participate in Watson–Crick base pairing interactions with the corresponding template base, and the RNAs are in posttranslocated states (Fig. 4).

Because the biochemical studies above showed that ara-NMP incorporation induced polymerase pausing and not termination, we also determined a structure with an incorporated ara-UMP and an incoming UTP (Figs. 3*C*, S7, S9). As a control, we also include a structure of polymerase that has incorporated two UMPs (Figs. 3*D*, S8, S9).

### Structural basis for poor SARS-CoV-2 polymerase ara-NTP selectivity over natural nucleotides

In both RNA and DNA polymerases, incoming nucleotides are recognized by a network of H-bonding and/or hydrophobic interactions with the 2′ and 3′ substituents of the ribose or deoxyribose sugar (1, 37, 38, 39). These 2′ and 3′ nucleotide substituents also influence the preferred pucker of the sugar where RNA bases favor a C3′-endo conformation and DNA bases prefer a C2′-endo conformation though nucleotides dynamically sample each conformation (40, 41). As the SARS-CoV-2 polymerase structure with incorporated ara-CMP also contains an incoming ara-CTP, we compared the incoming ara-CTP position to SARS-CoV-2 polymerase structures with natural incoming nucleotides such as our ara-UMP with incoming UTP structure or the published structures of 3′-deoxynucleotide terminated RNA with incoming CTP or UTP (30). In the ara-CMP–incorporated structure, the arabinose sugar of the incoming ara-CTP is poorly resolved with stronger density for base and triphosphate. The weaker density of the arabinose sugar than for the adjacent base and phosphate moieties implies heterogeneity in the sugar of the incoming ara-CTP, possibly owing to poor coordination by the polymerase NTP sugar–binding site (37, 42, 43). The incoming ara-CTP base makes Watson–Crick base pairing interactions with the templating base. The ara-CTP triphosphate has an altered position compared with incoming CTP (PDB code: 7UOE (30)) (Fig. S9), resulting in changes to the coordination of a polymerase catalytic site magnesium ion (metal B). In addition, despite the binding of an incoming nucleotide, the ara-CTP polymerase structure remains in an open polymerase state. It has previously been hypothesized that engagement with incoming nucleotide 2′ and 3′ hydroxyl groups results in active site closure prior to catalysis (37, 42). As the ara-UMP incorporated with incoming UTP structure adopts a closed precatalytic polymerase (Fig. 4), we assign the failure to close the active site for catalysis to the incoming ara-CTP rather than the incorporated ara-CMP. Together, the lack of stabilization for the binding of the arabinose sugar, an altered binding of the triphosphate to the polymerase catalytic center, and the failure to close the polymerase active site all likely contribute to poor competition of ara-NTPs with natural NTPs for incorporation into the growing RNA strand.

### Structural mechanism of action for ara-NMP–induced polymerase pausing

Despite not competing well with natural nucleotides, incorporation of ara-NMPs into nascent chains leads to strong polymerase pausing as described above in magnetic tweezer and bulk biochemistry experiments. A comparison of the ara-UMP– and ara-CMP–incorporated SARS-CoV-2 polymerase structures with published structures shows no large differences in either the protein or RNA components (Fig. S9). Positive-sense RNA polymerases undergo a multistep cycle to incorporate a nucleotide into nascent RNA. These include NTP binding, active-site closure, catalysis, active-site opening, and translocation (37, 43). Each of these steps could potentially be inhibited by nucleotide analog polymerase inhibitors. The structures with incorporated ara-CMP and ara-UMP have each adopted post-translocated states (Fig. 4), suggesting that for each nucleotide analog, the post-translocated state is favored at equilibrium and translocation is not strongly inhibited by ara-NMP incorporation. As shown in both bulk biochemistry and magnetic tweezer experiments, single incorporation of ara-NMPs is sufficient for polymerase pausing, ruling out a delayed pause in extension as a mechanism of polymerase inhibition. The polymerase active-site conformation with incorporated ara-NMP does not prevent incoming NTP binding as shown in the ara-UMP–incorporated structure with incoming UTP (Fig. 4). Moreover, the binding of the natural UTP in this structure promotes active site closure identically to other SARS-CoV-2 structures with the expected shifting of polymerase catalytic motif A to close the active site and UTP positioning identically to incoming natural nucleotides (30) (PDB code: 7UO9) (Fig. S9). Because the SARS-CoV-2 polymerase adopts a closed, catalytically active confirmation when bound to an incoming NTP (30, 37) but does not carry out catalysis, we infer that the catalysis itself is the rate-limiting step in extension of a primer with a terminal ara-NMP.

We assessed the ability of the SARS-CoV-2 polymerase to extend from a terminal dCMP and dUMP. In the treatment of leukemia, ara-CTP is used as an analog of dCTP to inhibit DNA polymerases (22) where ara-NMP incorporation stalls or terminates DNA synthesis (21, 44, 45). While both dCTP and dUTP are incorporated into primer RNAs by the SARS-CoV-2 polymerase, further extension of the primer using the remaining NTPs indicates a strong pausing of polymerase extension similar to that observed for ara-CTP and ara-UTP (Fig. S10).

### Ara-CTP and ara-UTP inhibition of poliovirus polymerase

To evaluate the extent to which arabinose nucleotides may broadly inhibit viral RNA polymerases, we tested the effects of both ara-CTP and ara-UTP against poliovirus RNA polymerase (Figs. S1*B*, S11). The IC_50_ for both ara-CTP and ara-UTP are 1000 μM in the presence of 1 μM CTP or UTP. To further measure the efficiency of incorporation, we determined the kinetic parameters: the *k*_pol_ value, the maximal rate constant for nucleotide incorporation, and the *K*_d,app_ value, the apparent dissociation constant for NTP binding, for each ara-NTP, and the corresponding natural nucleotide using a stopped-flow fluorescence apparatus (46, 47). The differences in efficiency between ara-CTP and ara-UTP and natural nucleotides are 50- and 90-fold lower, respectively (Table S3-4, Fig. S12).

We also assessed the action of ara-UTP and ara-CTP using our high-throughput magnetic tweezer assay. Differing from our magnetic tweezer assay for SARS-CoV-2 polymerase, the assay to assess poliovirus polymerase uses a dsRNA template with a short 3′ hairpin. Polymerase extension from the 3′ hairpin converts the dsRNA tether into ssRNA, increasing the length of the tether and shifting the position of the magnetic bead. The change in magnetic bead vertical position is then converted to nucleotides extended (48) (Figs. S13-14). Inclusion of ara-CTP or ara-UTP at ratios of 1:10 or 1:5 to their natural NTPs had very mild effects on pause rates or probabilities. Increasing the nucleotide to analog ratios to 1:1, we observe a threefold increase in ara-NTP pause probabilities for each analog (Tables S5-6).

## Discussion

The catalytic cycles of positive-sense viral RNA–dependent RNA polymerases have been extensively studied using both biochemistry and structural biology, and these cycles are believed to be shared among viral RNA–dependent RNA polymerases (37, 42, 43). These studies have proposed a six-step cycle. Starting from an RNA-bound ground state, an NTP binds the RNA polymerase in the +1 nucleotide position, base pairing with the template RNA. Nucleotide selection is based on networks of hydrogen bonding to conserved polymerase amino acids. The formation of these networks induces a closure of the active site where polymerase motif A moves into alignment with motif C and forming the magnesium-coordinated catalytic active site. Phosphoryl transfer occurs between the 3′ hydroxyl of the primer nucleotide and the α-phosphate of the incoming NTP, forming a new phosphodiester bond and pyrophosphate. The polymerase active site then reverses its conformational change to reopen the active site. Pyrophosphate dissociates, and the polymerase translocates along the RNA to allow the cycle to repeat.

Ara-CTP and ara-UTP induce strong pausing and inhibition of SARS-CoV-2 and poliovirus polymerases. We have shown that single ara-NTP incorporation is sufficient to induce long-lived pausing of SARS-CoV-2 polymerases. However, we observe a poor ability of ara-NTPs to compete with their corresponding natural NTPs likely owing to the ara-NTPs’ suboptimal interactions with polymerase active sites. The suboptimal interactions of the ara-NTPs may be due to altered base pairing or base stacking (33, 34) or poor polymerase coordination of the arabinose sugar. This suboptimal polymerase engagement may lead to the observed inefficiency of polymerase active site closure that precedes catalysis. We also observed that the triphosphate of incoming ara-CTP has an altered binding pose compared with CTP (PDB code: 7UOE (30)). However, the observed ara-CTP triphosphate interaction may be representative of the interactions of an NTP binding to the open polymerase state (37, 49).

In our biochemistry and magnetic tweezer experiments, incorporation of an ara-NMP causes the SARS-CoV-2 polymerase to pause. Our structural data show that the pausing is neither the result of poor RNA translocation, NTP binding, or active site closure nor is the polymerase active site distorted to prevent catalysis. We propose that polymerase pausing after ara-NMP incorporation may be due to an arabinose sugar conformation that is nonconducive for nucleophilic attack on an incoming NTP α-phosphate. B-form DNA is composed of C2′-endo sugars of deoxyribose, whereas A-form RNA is composed of C3′-endo ribose. However, these nucleotide sugars are believed to exist in a dynamic equilibrium between C2′-endo and C3′-endo conformers (40, 41). Time-resolved X-ray crystallography experiments of DNA polymerases have suggested that both the primer terminal nucleotide and the incoming nucleotide adopt C3′-endo conformers for catalysis in all polymerases (50).

Cryo-EM reconstructions of the SARS-CoV-2 polymerase are generally determined in a resolution range of 2.5 to 3.5 Å. To date, no determined SARS-CoV-2 polymerase structure, including those presented here, has sufficient resolution to unambiguously determine the sugar pucker of the primer terminal nucleotide or incoming nucleotide. Previous studies of DNA polymerases with incorporated or incoming ara-nucleotides determined by X-ray crystallography provide some insights into the sugar conformers of polymerase-bound ara-nucleotides (35, 36, 51). An X-ray crystal structure of DNA Pol-λ has an incorporated ara-CMP in a pretranslocated state, where both the −1 and +1 nucleotides adopt C3′-endo conformers. The observation of the C3′-endo conformers is expected for a nascent RNA that has just undergone phosphoryl transfer as noted above (51). In DNA Pol-η, an incoming ara-CTP adopts a C3′-endo conformer likely because of polymerase hydrogen bonding with the 2′ hydroxyl group. However, upon incorporation and translocation, ara-CMP adopts the C2′-endo conformer, and it was concluded from these structural data that the arabinose sugar promotes the C2′-endo conformer, inhibiting subsequent nucleotide incorporation (35). Among the RNA polymerases, there are no published structures with bound ara-NTPs. There are structures of the poliovirus RNA–dependent RNA polymerase that have been determined with 2′deoxy-CTP (dCTP). In both the precatalytic and postcatalytic polymerase states, the incoming dCTP adopts a C2′-endo conformer (37).

We propose that ara-CTP and ara-UTP inhibit viral polymerases by inducing a strong pause immediately after single nucleotide analog incorporation and translocation, driven by the C2′-endo sugar pucker conformation being nonconducive for nucleophilic attack on the α-phosphate of incoming NTPs. Both SARS-CoV-2 and poliovirus polymerases show similar long-lived pauses induced upon ara-CMP and ara-UMP incorporation. These data suggest that the adoption of C2′-endo sugar puckers by arabinose nucleotides may be a common mechanism of inhibition across polymerases and that nucleoside scaffolds with C2′-endo sugars may find use in the development of broad-spectrum RNA virus antivirals.

While the use of ara-nucleosides is an attractive scaffold for further discovery, significant improvements in the ara-NTPs studied here will be necessary to bypass several shortcomings in these nucleotide analogs prior to their therapeutic use. The steric clash between the ara-NTP 2′OH and the H6 pyrimidine base hydrogen (33, 34) may result in weaker base pairing with the template base and reduce base stacking leading to weakened binding of ara-NTPs to SARS-CoV-2 nsp12 NTP-binding site. 2′fluoro-ara-NTPs, which replace the 2′OH with a 2′F, should be evaluated against viral RNA polymerases as these analogs make pseudo-H-bonds to their own H6 rather than creating a steric clash (33, 34). This would preorganize the nucleotide base for base pairing with template nucleotides, which may partly overcome the observed issue with competition of ara-NTPs with natural NTP substrates. For reasons that remain unclear, the ara-CTP appears to compete better with CTP than ara-UTP competes with UTP *in vitro*, and intracellular CTP levels are typically much lower than other nucleotides (52). We therefore suggest the development of ara-CTP as a better scaffold for elaboration than ara-UTP.

There are also cytotoxicity concerns regarding the use of ara-NTPs as antiviral drug scaffolds because ara-cytidine, the nucleoside of ara-CTP, is used clinically to treat leukemia. Ara-cytidine is converted into ara-CTP in cells and exerts its anticancer effects by competing with dCTP for incorporation into DNA with Pol-α and Pol-ε (21, 23). Like its effect on the viral polymerases studied here, incorporated ara-CMP is extended poorly by DNA polymerases leading to replication stress and cell death through the stalling of replication forks and the creation of double-stranded DNA breaks (23). The development of nucleotide analogs that target the SARS-CoV-2 replication machinery has been difficult because of the proofreading activity of the viral exonuclease. While we do not evaluate the efficacy of ara-nucleosides in reducing SARS-CoV-2 titers and do not test the ability of the viral exonuclease to remove ara-NTPs from RNA termini, proofreading of incorporated ara-NMPs has been examined in several DNA polymerases, and the ara-NTP analogs are efficiently excised by those polymerases possessing 3′ to 5′ exonuclease activity (21, 23). The excision of the nucleotide from nascent RNA in SARS-CoV-2 replication would be expected to narrow the therapeutic window in which these analogs could be used as drugs.

In summary, we present ara-NTPs as a scaffold for further elaboration and development of novel antiviral drugs targeting diverse RNA viruses. We propose that the adoption of a C2′-endo sugar pucker in incorporated ara-NMPs induces long pausing in viral polymerases and identify several limitations of the current ara-NTPs that must be overcome prior to their deployment as therapeutics. The introduction of scaffolds for viral polymerase inhibition and preliminary characterization of those scaffolds’ mechanisms of inhibition will be essential to address evolving viral threats both from existing and emerging viruses.

## Experimental procedures

### Reagent production

SARS-CoV-2 nsp7, nsp8, and nsp12 were expressed and purified as previously described (53). Briefly nsp7 and nsp8 were expressed in *Escherichia coli*, whereas nsp12 was produced using the baculovirus expression system. Proteins were purified from clarified supernatants using affinity chromatography and size-exclusion chromatography (Supporting Methods).

Poliovirus polymerase, 3D^pol^, was expressed and purified as previously described (54, 55). Briefly, poliovirus 3D^pol^ polymerase was expressed as an N-terminal ubiquitin fusion in *E*. *coli*. Proteins were purified using affinity chromatography, cation exchange, and size exclusion chromatography. Ubiquitin was removed during purification to create a native N terminus on poliovirus 3D^pol^ (Supporting Methods).

Large RNAs for magnetic tweezer experiments were produced as previously described (56). Briefly, the RNAs were produced as fragments and then ligated together producing a 1 kb RNA for SARS-CoV-2 polymerase experiments and a 2.8 kb double-stranded RNA for poliovirus polymerase experiments (Supporting Methods).

Arabinose nucleotides were purchased from Jena Bioscience.

### SARS-CoV-2 and poliovirus-catalyzed primer extensions with detection of ^32^P-labeled products

Reactions to assess the incorporation of ara-NTPs by single and double nucleotide incorporations combined SARS-CoV-2 polymerase components: 0.5 μM nsp12, 1.5 μM nsp7, 1.5 μM nsp8, or 1 μM poliovirus 3D^pol^ polymerase enzyme, with 1 μM annealed synthetic RNA primer–template pairs, 100 μM ATP, and 0.1 μCi/μl [α-^32^P]-ATP and were incubated at 30 °C. Single nucleotide incorporation assays for SARS-CoV-2 polymerase were initiated by adding either 0.1 μM CTP or UTP, or 1 mM of ara-CTP or ara-UTP, and allowed to proceed for 10 min before quenching with 50 mM EDTA. Single nucleotide incorporation assays for poliovirus polymerase were initiated by adding either 1 μM CTP or UTP or 1 mM ara-CTP or ara-UTP and allowed to proceed for 10 min before quenching with 50 mM EDTA. For chain termination experiments for each polymerase, reactions were performed as above in the presence of the next correct nucleotide substrate (1 μM UTP or 1 μM CTP), followed by quenching with 50 mM EDTA.

To evaluate the nucleotide preference of the SARS-CoV-2 polymerase complex for ara-CTP or ara-UTP, polymerase–RNA elongation complexes were assembled, and the reactions were initiated with 0.1 μM CTP or UTP and increasing concentrations of competing ara-CTP or ara-UTP, respectively. These reactions were incubated for 10 min along with next correct nucleotide (1 μM UTP or 1 μM CTP) and then quenched with 50 mM EDTA.

Reaction products were separated by 20% denaturing urea-PAGE, analyzed by phosphorimaging and quantitated. Reactions were repeated three to four times. Data were fit by either linear or nonlinear regression using GraphPad Prism, version 7.03 (GraphPad Software, Inc).

### High-throughput magnetic tweezers for examining viral polymerase activity

For SARS-CoV-2 polymerase extensions, 0.1 ng of ssRNA template was bound to streptavidin-coated magnetic beads (ThermoFisher), and excess RNA was washed away. Magnetic beads bound to ssRNA templates were bound to flow cells and flushed with reaction buffer (50 mM Hepes [pH 7.9], 10 mM DTT, 2 μM EDTA, and 5 mM MgCl_2_). The flow cell was then flushed with reaction buffer containing 0.6 μM SARS-CoV-2 nsp12, 1.8 μM nsp8, 1.8 μM nsp7, NTPs, and ara-CTP as indicated. Reactions were run at a constant force for 30 to 60 min. For poliovirus polymerase extensions, 0.1 ng of dsRNA template was bound to streptavidin-coated magnetic beads. The beads were then washed, and the complexes were bound to flow cells. Flow cells were rinsed in reaction buffer (50 mM Hepes [pH 7.9], 5 mM MgCl_2_, 0.01% Triton X-100, 5% SUPERase RNase inhibitor ([Life Technologies]). The flow cells were then flushed with reaction buffer containing 1.44 μM poliovirus polymerase, NTPs, and ara-NTP as indicated. Reactions were run at a constant force for 60 to 75 min.

Bead displacement was converted to nucleotide progression using a linear interpolation formula. Traces were analyzed using a dwell time analysis, where traces are separated into nonoverlapping 10-nucleotide windows. A maximum-likelihood estimation was used to analyze all the dwell times for a particular experimental condition and extract the parameters for a stochastic pausing model of polymerase extension (27) (Supporting Methods).

### Fluorescence-based RNA primer extension for incorporation and chase

To examine the extension from incorporated nucleotide analogs, an RNA primer–template pair was bound to the SARS-CoV-2 polymerase, and a single ara-NTP or dNTP was incorporated into the fluorescently labeled primer. The reaction was then chased with 40 μM of each of the remaining NTPs, and reactions were followed over a time course to examine the formation of the full-length RNA product by denaturing urea-PAGE (Supporting Methods).

### Single-particle cryo-EM for structure determination

SARS-CoV-2 nsp7, nsp8, and nsp12 were combined with an annealed RNA primer–template pair, and nucleotides or nucleotide analogs were additionally added and incubated with the complex. The ara-CTP sample had a final protein concentration of 4 mg/ml, whereas the ara-UTP and UTP samples had final protein concentrations of 8 mg/ml. Samples were mixed with CHAPSO detergent just before preparing grids. Samples were spotted onto UltrAuFoil grids (Quantifoil) and plunge frozen in liquid ethane. Data were collected on a Talos Arctica (ThermoFisher) using a K3 direct electron detector (Gatan). Micrograph movies were processed in CryoSPARC (57) to produce reconstructed density maps, and coordinate models were produced using COOT (58), PHENIX (59) and ISOLDE (60) using 7UOE.pdb (30) as a starting model (Supporting Methods).

### Poliovirus 3D^pol^ polymerase stopped-flow experiments

Presteady state nucleotide incorporation for ara-CTP, CTP, ara-UTP, and UTP by the poliovirus polymerase was determined by changes in RNA template analogs’ fluorescence because of nucleotide incorporation into symmetrical RNA substrates and monitored by a stopped-flow apparatus. Using fluorescence traces from reaction time courses containing a range of substrate concentrations allowed determination of the poliovirus polymerase apparent dissociation constant, K_d,app_, and polymerization rate constant, k_pol_, for each nucleotide (Supporting Methods).

## Data availability

Structure coordinate data and reconstructed maps are available for download at the PDB or the Electron Microscopy Data Bank. The ara-CMP and ara-CTP structure has accession numbers pdb_00009BLF, EMD-44654. The ara-UMP structure has accession numbers pdb_00009PYW, EMD-72038. The ara-UMP and UTP structure has accession numbers pdb_00009PYZ, EMD-72053. The structure with two incorporated UMP has accession numbers pdb_00009PZ0, EMD-72054.

## Supporting information

This article contains supporting information(61, 62, 63, 64, 65).

## Conflict of interests

The authors declare that they have no conflicts of interest with the contents of this article.
